# Downregulation of the Adenosine A2b Receptor by RNA Interference Inhibits Hepatocellular Carcinoma Cell Growth

**DOI:** 10.5402/2011/875684

**Published:** 2011-04-28

**Authors:** Hong-Jun Xiang, Fu-Lu Chai, De-Sheng Wang, Ke-Feng Dou

**Affiliations:** Department of Hepatobiliary Surgery, Lanzhou General Hospital, Lanzhou Military Area Command of Chinese People's Liberation Army, 98 XiaoXiHu Road, Lanzhou 730050, China

## Abstract

To investigate the biological effect of adenosine A2b receptor (A2bR) on the human hepatocellular carcinoma cell line HepG2, three A2bR siRNA constructs were transiently transfected into HepG2 cells. The results showed that A2bR siRNA reduced the levels of A2bR mRNA and protein. In order to further detect the function of A2bR, we established a stable hepatocellular carcinoma cell line (HepG2) expressing siRNA targeting the adenosine A2b receptor. Targeted RNAi significantly inhibited tumor cell growth *in vitro*, and flow cytometry (FCM) showed that significantly more cells expressing A2bR siRNA were in the G0/G1 phase compared to the untransfected group ((89.56% ± 3.15%) versus (56.19% ± 1.58%), *P* < 0.01). These results indicated that silencing the expression of adenosine A2b receptor in HepG2 cells can suppress cell growth effectively by blocking the cell cycle. Downregulation of adenosine A2b receptor gene expression with RNA interference could be a new approach to hepatocellular carcinoma therapy.

## 1. Introduction

Adenosine is an intermediate product of adenine nucleotide metabolism. In many organs, adenosine is released into the extracellular space when the oxygen supply is decreased or energy consumption is increased [1, 2]. In particular, significant levels of adenosine are found in the extracellular fluid of solid tumors, suggesting a role for adenosine in tumor growth [3]. Adenosine is an endogenous nucleoside that modulates many physiological processes through its interaction with at least four membrane receptors: A1, A2a, A2b, and A3 [4, 5]. The A2 receptors are divided into two subtypes: high-affinity A2 receptors in rat striatum and low-affinity A2 receptors throughout the brain [6]. These high- and low-affinity receptor subtypes were later designated as A2a and A2b, respectively [7]. The knowledge of A2b receptors lags behind that of other receptor subtypes. It was thought that the A2b receptor plays a small role *in vivo* because of its relatively low affinity for adenosine. Recently, Spychala discovered that activation of adenosine receptors may be involved in tumor progression [8]. 

In a previous study, we collected 64 samples of hepatocellular carcinoma from clinical patients and found that A2b receptors were highly expressed in tumor tissue and expressed in peritumoral tissues to a lesser extent by real-time PCR, Western blot, and immunohistochemical staining. These results indicate that the A2b receptor may contribute to hepatic tumor progression and may represent a good therapeutic target [9].

In recent years, RNA interference (RNAi) has been widely used to study gene expression regulation in mammalian cells and therapeutic intervention for various diseases including cancer [10]. In this study, we investigate whether silencing the A2B gene has an effect on hepatocellular carcinoma.

## 2. Materials and Methods

### 2.1. Vector-Based shRNA Plasmid Constructs

Our vector is based on the pSilencer 3.1-H1 neo (*Hin*d III/*Bam*H I) vector (Ambion) which contains the human H1 promoter and neomycin resistance gene to enable antibiotic selection in mammalian cells. Three pairs of complementary oligonucleotides (shRNA1, shRNA2, and shRNA3) were synthesized, targeting adenosine A2b receptor cDNA (GenBank: NM_000676*；*CDS 333–1331) at nucleotides 591–610, 856–875, and 909–928, respectively. The oligonucleotides encoding the human adenosine A2b receptor shRNA targeting the adenosine A2b receptor were as follows (http://www.ambion.com/techlib/resources/RNAi/index.html).

**shRNA 1**

5′-GATCCCGTGCTGGTGATCTACATTAATTCAAGAGATTAATGTAGATCACCAGCATTTTTTGGAAA-3′
5′-AGCTTTTCCAAAAAATGCTGGTGATCTACATTAATCTCTTGAATTAATGTAGATCACCAGCACGG-3′

**shRNA 2**

5′-GATCCCGTCCCATTGTCTATGCTTACTTCAAGAGAGTAAGCATAGACAATGGGA TTTTTTGGAAA-3′
5′-AGCTTTTCCAAAAAATCCCATTGTCTATGCTTACTCTCTTGAAGTAAGCATAGACAATGGGACGG-3′

**shRNA 3**

5′-GATCCCGTTATCTCCAGGTATCTTCTTTCAAGAGAAGAAGATACCTGGAGATAATTTTTTGGAAA-3′
5′-AGCTTTTCCAAAAAATTATCTCCAGGTATCTTCTTCTCTTGAAAGAAGATACCTGGAGATAACGG-3′

All oligonucleotides were synthesized by SBS Genetech, Ltd. The synthesized shRNA cassette was annealed and cloned into the pSilencer3.1-H1 neo vector according to the manufacturer's instructions. A BLAST search with the target sequences was performed to ensure that only the adenosine A2b receptor gene was targeted. A scrambled control plasmid (pSilencer-3.1-Y) encoding a shRNA contained a sequence not present in the mouse, human, or rat genome databases. The sequence was as follows.

**shRNA**

5′-GATCCAGTTCAACGACCAGTAGTCTTCAAGAGAGACTACTGGTCGTTGAACTTTTTTTGGAAA-3′ and
5′-AGCTTTTCCAAAAAAAGTTCAACGACCAGTAGTCTCTCTTGAAGACTACTGGTCGTTGAACTG-3′.

### 2.2. Plasmid Transfection and Establishment of Stable-Expression Models of Adenosine A2b Receptor in HepG2 Cells

HepG2 cells were cultured in Dulbecco's modified Eagle's medium (DMEM, Gibco), supplemented with 10% heat-inactivated FBS in a humidified incubator with 5% CO_2_ at 37°C. Cells were transfected with Lipofectamine 2000 (Invitrogen) according to the manufacturer's instructions with 8 *μ*g pSilencer 3.1-H1 neo-mix (shRNA1, shRNA2, and shRNA3) and pSilencer-3.1-Y. One day after transfection, cells were screened in medium containing G418 (0.4 mg/mL) and resistant clones were maintained in medium containing 0.4 mg/mL G418. Stably transfected cells were plated in 60-mm dishes (1.5 × 10^6^ per dish). The results were observed under the microscope.

### 2.3. RT-PCR

The treated cells were trypsinized and then centrifuged for 2 minutes at 12,000 rpm at 4°C. Cell pellets were washed with PBS, collected, and lysed with TRIzol reagent (Gibco). Total RNA was isolated by phenol/chloroform extraction, isopropanol precipitation, and 75% ethyl alcohol wash and dissolved in DEPC water. The reverse transcription reaction was set up according to Promega's Reverse Transcription System protocol using primers for the A2b receptor (forward primer: 5′-TCCATCTTCAGCCTTCTGGC-3′; reverse primer: 5′-AAAGGCAAGGACCCAGAGGA-3′) and *β*-actin (forward primer: 5′-GCCCTGAGGCACTCTTCCA-3′; reverse primer: 5′-GAAGGTAGTTTCGTGGATGCCA-3′). Each PCR reaction was performed using an optimized number of cycles (25 cycles) of 94°C for 1 minute, 56°C for 30 seconds, and 72°C for 40 seconds, with a final extension of 72°C for 7 minutes. The PCR reactions were visualized on a 1.2% agarose gel containing 5 *μ*g/mL of ethidium bromide. Intensity of the DNA bands was analyzed by Glyko BandScan Analysis software, using *β*-actin as an internal standard.

### 2.4. Western Blot

Cell monolayers in six-well culture plates were washed twice with ice-cold PBS and lysed with 150 *μ*L of RIPA lysis buffer (0.05 M Tris-HCl (pH 7.4), 0.15 M NaCl, 0.25% deoxycholic acid, 1% NP-40, and 1 mM EDTA) containing protease inhibitors (1 mM PMSF, 1 *μ*g/mL aprotinin, and 1 *μ*g/mL leupeptin). Protein concentration was measured (BCA Protein Assay, Pierce), and samples were resolved by reducing SDS-PAGE, transferred to nitrocellulose, and incubated in blocking buffer (25 mM Tris-HCl, pH 8.0, 125 mM NaCl, 0.05% Tween 20, and 5% BSA). Membranes were incubated with an antibody to the adenosine A2b receptor (Chemicon International, diluted 1 : 200) at 4°C overnight. The membranes were washed in blocking solution and incubated with an HRP-conjugated secondary antibody for 1 hour at room temperature. Detection was performed using the Pierce chemiluminescence HRP substrate according to manufacturer's instructions. Blots were exposed to X-ray film for the appropriate time period.

### 2.5. Soft Agar Colony Formation Assay

A total of 2 × 10^2^ cells/well were suspended in complete medium containing 0.3% agarose (Gibco) and seeded in triplicate in six-well plates onto a bottom layer of complete medium containing 0.6% agarose. The plates were cultured for 14 days. Colonies were counted after methanol fixation and Giemsa staining.

### 2.6. Flow Cytometry

A total of 50,000 cells were used for fluorescence-activated cell sorter analysis (FACS). Briefly, the cells were harvested, washed twice with PBS buffer, and then incubated at room temperature for 45 minutes. After two PBS washes, the cells were resuspended in PBS and filtered through Spectra Mesh filters (Spectrum). Data were analyzed by CellQuest software (Becton-Dickinson) and the ModFit/LT software. At total of 20,000 events were acquired from every sample.

### 2.7. MTT Assay

HepG2 cells were passed into 96-well plates (1,000 cells/well) 24 hours after transfection. Viability and proliferation of cells were measured using MTT assays from the second until the seventh day after passage. For each group, 6 wells were measured every day. Experiments were carried out a total of three times, and the difference in average OD (490 nm) between treated groups was compared using the unpaired, two-tailed *t*-test.

### 2.8. Statistical Analysis

Statistical analyses were performed with SPSS11.0 software. Significance was evaluated by Student's *t*-test for paired or independent samples. Results were expressed as mean ± standard error (SE). Values with *P* < 0.05 were considered significant.

## 3. Results

### 3.1. Plasmid Construction

**Figure 1:**
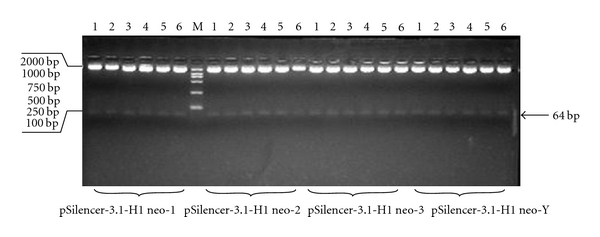
Plasmid construction. The construction of vectors pSilencer-3.1-H1 neo-1, pSilencer-3.1-H1 neo-2, pSilencer-3.1-H1 neo-3, and pSilencer-3.1-H1 neo-Y.* Hin*d III and *Bam* H I were used to digest the vector, and a 64 bp fragment was excised.

Four pairs of primers were cloned into pSilencer3.1-H1 neo (*Hin*d III/*Bam*H I) vector to construct four interfering vectors: pSilencer3.1-H1 neo-1, pSilencer3.1-H1 neo-2, pSilencer3.1-H1 neo-3, and pSilencer3.1-H1 neo-Y. Plasmids were verified by sequencing (Figure 1).

### 3.2. Suppression of Adenosine A2b Receptor Expression by the siRNA Vector

**Figure 2:**
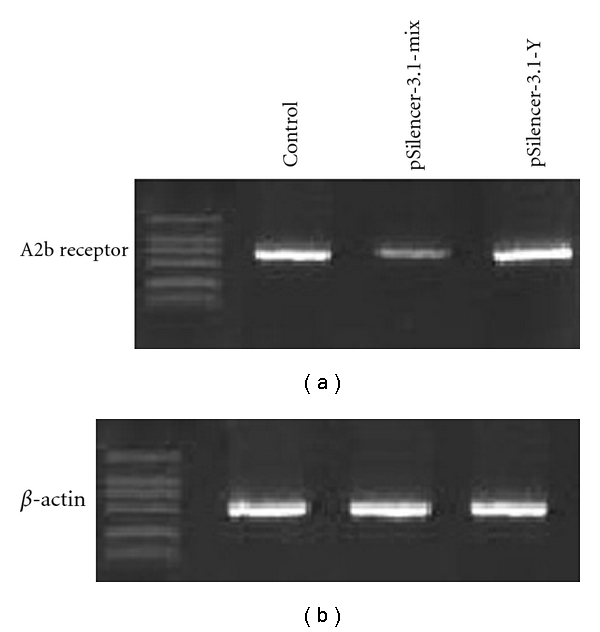
mRNA expression levels of A2b receptor after transient transfection of the Mix siRNA constructions into HepG2 cell lines by RT-PCR. Compared to normal controls, transfection with pSilencer3.1-mix significantly reduced the mRNA of the A2b receptor. The pSilencer3.1-Y did not affect the mRNA level of A2b receptor. *β*-actin was amplified to verify equal loading.

To verify the silencing effect of the three siRNA vectors, we transfected the three vector pSilencer3.1-H1 neo-1, 2, 3 (mixed) and pSilencer3.1-H1 neo-Y (as control) into the HepG2 cell line. RT- PCR showed that transfection of mixed vector could decrease the A2b receptor mRNA about 66% ± 5%, while pSilencer3.1-H1 neo-Y had no effect (Figure 2). 

**Figure 3:**
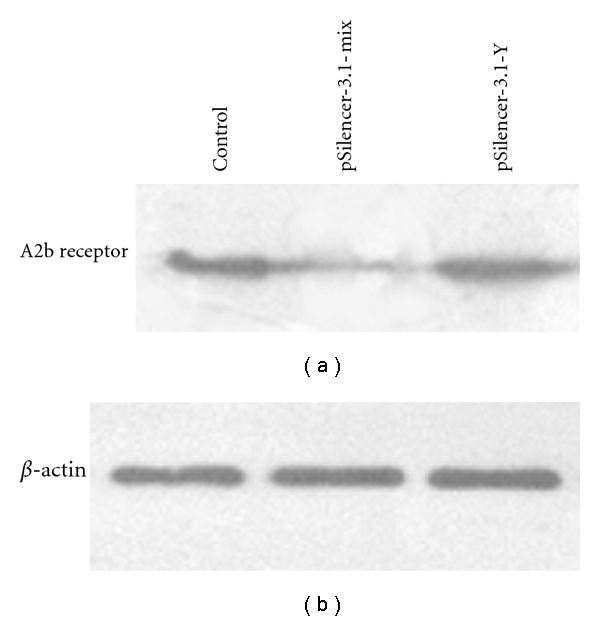
Protein expression levels of A2b receptor after transient transfection of the mixed siRNA constructs into HepG2 cell lines. Compared to normal controls, the pSilencer3.1-mix significantly reduced protein expression of the A2b receptor. The pSilencer3.1-Y did not affect the protein level of A2b receptor. *β*-actin was detected to verify equal loading.

Western blot analysis of A2b receptor was comparable to the RT-PCR results (Figure 3). Cells transiently transfected with pSilencer3.1-H1 neo-1, 2, 3 decreased levels of adenosine A2b receptor (about 70% ± 8%). Cells transfected with pSilencer3.1-H1 neo-Y had no effect on A2b receptor protein levels.

### 3.3. Transfection of pSilencer3.1-neo-mix Inhibits HepG2 Cell Growth

**Figure 4:**
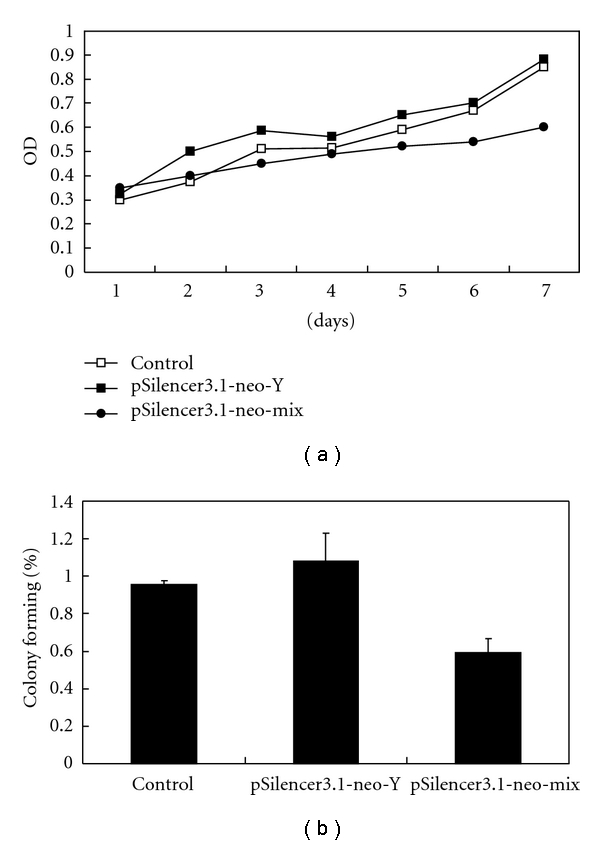
Inhibition of cell growth by stable transfection of pSilencer3.1-neo-mix vectors. (a) Anchorage-dependent cell growth was determined by MTT assay. HepG2 cells transfected with pSilencer3.1-neo-mix constructs inhibits proliferation. The *x*-axis indicates cultured time period, and the *y*-axis represents the row OD value. Growth rate of cells transfected with control vector (pSilencer3.1-neo-Y) or untransfected cells was compared. (b) Anchorage-independent cell growth was measured by the soft agar assay. Data are presented as means ± SEM.

To assay the potential role of A2b receptor, HepG2 cells were stably transfected. MTT assays were used to investigate anchorage-dependent growth rates. Equal amounts of control vector pSilencer3.1-neo-Y were transfected into HepG2 cell lines. Untransfected cells were used as an additional control. After seven days in culture, we compared the proliferation rate of the three groups of cells. A significant difference was observed after five days in culture. The cells transfected with pSilencer3.1-neo-mix showed a significant decrease in the proliferation rate after five days in culture. However, cells transfected with pSilencer3.1-neo-Y and untransfected cells had similar proliferation rates (Figure 4(a)). 

To determine anchorage-independent growth rates, soft agar assays were carried out. Control-transfected cells showed an average colony forming efficacy of 108 ± 10.7%, compared to untransfected HepG2 cells (= 100%). In contrast, stably transfected cells showed a decreased colony forming ability of 60.33 ± 9.6% compared with HepG2 cells (*P* < 0.05) (Figure 4 (b)).

### 3.4. Transfection of pSilencer3.1-neo-mix Induces Cell Cycle Arrest of HepG2

**Figure 5:**
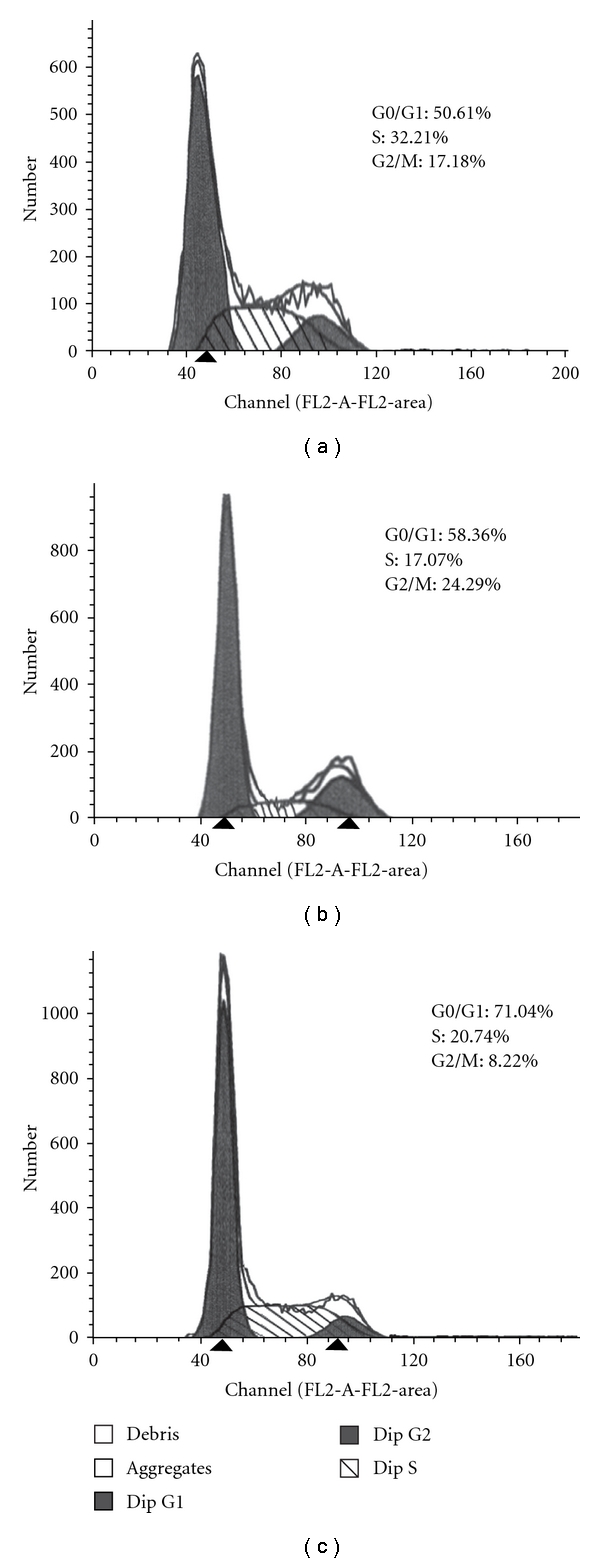
Analysis of cell cycle after silencing the A2b receptor gene in HepG2 cell line. Stably transfected HepG2 cells were prepared for FACS analysis after 5 days. The pictures show one representative experiment from three total repeats.

To explore the mechanism of growth suppression in A2bR-silencing cells, cell cycle analysis was performed. The number of cells in Go/G1 phase was 89.56, 62.01, and 56.19% for the pSilencer3.1-neo-mix, pSilencer3.1-neo-Y, and untransfected cells, respectively (Figure 5 and Table 1).

## 4. Discussion

Liver cancer is the fifth most important cancer worldwide in terms of morbidity but the third in terms of mortality. In addition, its mortality has increased in recent years, with 548,600 cases in the year 2000 [11]. Little is known about the mechanisms of hepatocarcinogenesis which seem to differ according to the risk factors involved. Many genes are associated with liver cancer.The p53 protein is involved in cell cycle control, senescence, DNA repair, genomic stability, and apoptosis, and different mutations of p53 are involved in hepatocellular carcinoma formation [12]. The pRB protein is phosphorylated during the G1 phase of the cell cycle by members of the cyclin-dependent kinase (CDK) system, and abrogation of the p16-Rb pathway has been observed in hepatocellular carcinoma [13]. The mutation of other genes, including *β*-catenin [14], SMAD2, SMAD4 [15], p16 INK4a [16], and Cyclin D1 [17], may also be implicated in liver cancer. 

Adenosine is an endogenous nucleoside that modulates many physiological processes. Its actions are mediated by interaction with specific cell membrane receptors. Adenosine receptors modulate intracellular levels of adenosine 3′,5′-cyclic monophosphate (cAMP). Significant advances have been made in the understanding of the molecular pharmacology and physiological relevance of adenosine receptors, but little is known about A2b receptors. A2b receptors have been implicated in mast cell activation [18] and asthma [19], vasodilation [20], regulation of cell growth [21], intestinal function [22], and modulation of neurosecretion [23]. In a previous study, we found that A2b receptors were highly expressed in hepatocellular carcinoma tissue, indicating that expression of the A2b receptor may contribute to tumor progression and may be a good therapeutic target for liver cancer.

RNA interference (RNAi) is a natural silencing process first described in *Caenorhabditis elegans *and *Drosophila melanogaster *[24] by which double-stranded RNA initiates and directs the degradation of homologous mRNA [25]. Specific inhibition of cellular mRNA by RNAi can be triggered in mammalian cells by the introduction of synthetic 21- to 23-nucleotide double-stranded small interfering RNA (siRNA) [26, 27] or alternatively by the transcription of siRNA from a DNA construct driven by the RNA polymerase cassette [28]. RNAi technology has been widely used as an extremely powerful strategy for reverse functional genomics [29, 30] and as an effective method for gene silencing-based therapeutics [31, 32]. RNAi-mediated inhibition of virus infection or replication has been reported for numerous viruses, including several important human pathogens such as HIV-1, hepatitis C virus, and Dengue virus [33–35]. RNAi-mediated oncogene silencing can also confer resistance to tumorigenesis [36].

In this study, we constructed three RNAi vectors which target the A2b receptor gene to investigate if silencing can convert the tumor phenotype. First, the mixed plasmid constructs were transiently transfected into HepG2 cells. As shown by RT-PCR and Western blot, transfected cells showed specific silencing of the A2b receptor gene without interrupting other molecular interactions. A stably transfected HepG2 cell line which silences the expression of A2b receptors was constructed to observe any changes in phenotype. MTT and soft agar assays show that the proliferation rate of the stably transfected cells was significantly decreased compared with control-transfected or untransfected HepG2 cells. FCM experiments revealed that 89.56 ± 3.15% of stably transfected cells were in the G1 phase. Taken together, these results show that RNAi silencing of the A2b receptor gene alters the phenotype of HepG2 cells, and this method might lead to development of novel therapies for the human hepatocellular carcinoma.

## Figures and Tables

**Table 1:** Cell cycle distribution showing the percentage of cells in G0/G1, S and G2/M phase according to DNA content.

Group	*N*	G0/G1 (%)	S (%)	G2/M (%)
pSilencer3.1-neo-Mix	3	89.56 ± 0.15	8.28 ± 0.95	2.16 ± 1.20
pSilencer3.1-neo-Y	3	62.01 ± 2.18	29.58 ± 2.31	8.41 ± 0.35
Control	3	56.19 ± 1.58	38.41 ± 5.92	5.40 ± 4.82
